# An Interdisciplinary Study Regarding the Characteristics of Dental Resins Used for Temporary Bridges

**DOI:** 10.3390/medicina58060811

**Published:** 2022-06-16

**Authors:** Ioana Mârțu, Alice Murariu, Elena Raluca Baciu, Carmen Nicoleta Savin, Iolanda Foia, Monica Tatarciuc, Diana Diaconu-Popa

**Affiliations:** 1 Department of Oral Implantology, Discipline of Dental Technology, Faculty of Dental Medicine, University of Medicine and Pharmacy “Grigore T. Popa”, 700115 Iasi, Romania; ioana.martu@umfiasi.ro (I.M.); monica.tatarciuc@umfiasi.ro (M.T.); antonela.diaconu@umfiasi.ro (D.D.-P.); 2 Department of Surgery, Discipline of Community Dentistry, Faculty of Dental Medicine, University of Medicine and Pharmacy “Grigore T. Popa”, 700115 Iasi, Romania; alice.murariu@umfiasi.ro; 3 Department of Oral Implantology, Discipline of Dental Materials, Faculty of Dental Medicine, University of Medicine and Pharmacy “Grigore T. Popa”, 700115 Iasi, Romania; 4 Department of Surgery, Discipline of Pediatric Dentistry, Faculty of Dental Medicine, University of Medicine and Pharmacy “Grigore T. Popa”, 700115 Iasi, Romania; 5 Department of Preventive Medicine and Interdisciplinarity, Discipline of Hygiene, Faculty of Dental Medicine, University of Medicine and Pharmacy “Grigore T. Popa”, 700115 Iasi, Romania; iolanda.foia@umfiasi.ro

**Keywords:** dentistry, temporary dental bridges, polymethyl methacrylate resin, surface characteristics

## Abstract

*Background and Objectives:* The surface condition of the materials that are used for temporary prostheses influences their microbial colonization, with a direct impact on the oral tissues. This study aims at a comparative analysis of three types of resins for temporary bridges using conventional and digital technologies. The attention was focused on the analysis of the surface characteristics and mechanical strength of these materials. *Materials and Methods:* The surface condition was assessed for three distinct materials both before and after polishing- heat-curing resin Superpont C + B (SpofaDental, Jicin, Czech Republic) used unconventional technology, Zotion dental milling polymethyl methacrylate (PMMA) block (Zotion, Chongqing, China) for provisional crowns/bridges used in digital subtractive technologies and Freeprint Temp (Detax GmbH & Co. KG, Ettlingen, Germany) resin for temporary crowns and bridges that are used in 3D printing technologies. The two-way ANOVA analysis indicated that polishing leads to a statistically significant increase in roughness coefficients for all the three resins that were tested (*p* < 0.001). While the highest roughness coefficients were displayed in the 3D cured sample, the largest decrease was reported by the milled sample *Results:* The results revealed that surface roughness was significantly influenced by both the type of resin that was used (*p* < 0.001) and the treatment that was induced by finishing and polishing (*p* < 0.001). Similar *p*-values were obtained for each of the three resins. *Conclusions:* The results demonstrated a significant optimization of the surfaces after finishing and polishing and statistically significant differences between the surface parameters and the mechanical properties of the samples. The low values of the roughness and the acceptable values of the mechanical resistance for the conventional samples indicate these materials for the long-term temporary bridge’s realization, allowing the correct restoration of the functions and the rehabilitation at the oral level.

## 1. Introduction

A well-planned therapeutic strategy which could be adapted to the clinical situation is essential for the long-term outcome of prosthetic rehabilitation. Incorrect prostheses may contribute to the progression of periodontal disease because there is a strong association between prosthetics and periodontics, as periodontal health has an important role in the longevity of fixed dental restorations [1,2].

To prevent functional disorders and the occurrence of local and loco-regional complications following the abutment preparation, the treatment plan should include a temporary prosthesis, realized by different methods and materials depending on the purpose and the period of applicability of these types of prosthetic devices [3].

The abutments preparation creates an imbalance both at the dental and periodontal level and by eliminating the contact points, there is a risk of occlusal disorders and temporomandibular joint dysfunctions [4].

To prevent these inconveniences, it is necessary to apply an interim prosthetic treatment, which aims at the following objectives: mechanical protection and position preservation of the abutments, restoration of occlusal contacts, directing the healing of periodontal tissues, and the maintenance of their position to the teeth. All these prevent bacterial colonization and ensures functional rehabilitation of the stomatognathic system, for the rapid social reintegration of the patient [5,6].

The marginal adaptation of the fixed prosthetic constructions is conditioned by the design of the abutments at the cervical level and preservation of this area can only be achieved with the help of the correct temporary prostheses. In addition, the surface condition of the materials that are used for interim devices influences microbial colonization, with a direct impact on the health of the periodontal tissues. The surface characteristics are influenced by the material properties and the technology which is used, especially by the final steps: finishing and polishing [7,8].

The materials that are involved in making temporary bridges must have several characteristics: dimensionally stable, resistant to functional and occlusal load, reasonably resistant to fractures, they should have sufficient compressive and flexural strength, tissue-friendly, shaped to promote healing and discourage plaque development, esthetically pleasing, proper functional restoration, relatively low price, and easy to adapt in the oral cavity. The technique should also ensure quick and simple fabrication [9,10,11].

The surface of temporary bridges must be rigorously prepared to be as smooth as possible in order to limit bacterial colonization. Successful tissue response to any composite material depends on several factors, and surface roughness, an important variable, can be significantly altered if the restoration is incorrectly finished and polished.

Improperly constructed “interim” restorations may cause periodontal inflammation and gingival recession and the requirements for fit, polish, and contour in the interim restoration should be the same as for the final restoration. Sometimes, inappropriate prosthetic treatment planning or the prosthesis itself can cause periodontitis or gingival recession [12].

The temporary restoration of the dental arches requires the choice of material and techniques that simplify the clinical and technological workflow as much as possible.

The conventional materials for provisional restorations care can be divided into two groups according to their chemical composition: those that are based on monomethacrylates or acrylic resins, which include polymethylmethacrylate (PMMA) and polyethyl/butyl methacrylate (PEMA); and those that are based on dimethacrylates or bis-acryl/composite resins such as bisphenol A-glycidyl dimethacrylate (Bis-GMA) and urethane dimethacrylate (UDMA) [13]. These materials, used in conventional technologies, can also have a number of disadvantages such as low mechanical strength, color instability, the existence of residual monomer [14,15,16,17,18].

For several decades, polymethyl methacrylates have been considered as the standard. Their advantages include low cost, high strength, polishability, color stability, as well as easy repair, reline, and adjustments. Disadvantages of these materials include high exothermic reaction which may traumatize the pulp, release of residual monomers causing tissue damage and 20% incidence of allergic sensitivity, relatively high polymerization shrinkage, objectionable odor, and potential variations when hand mixing powder and liquid [19,20].

Different types of materials are prone to produce effects which can affect the final product in terms of shrinkage by polymerization. Volumetric and surface changes of resins that are used for interim fixed prosthesis can influence the elements of the prosthetic area, with negative consequences on the final therapeutic solution [21,22,23]

Digital systems that are used to build temporary bridges, such as computer-aided design/computer-aided manufacturing (CAD/CAM) milling and 3D-printing, greatly reduce workflow and can be used in both direct and indirect technologies [24].

Comparing interim restorations that were fabricated by CAD/CAM with those that were conventionally made had several aspects that were taken into account: color stability, water sorption, wear resistance, surface hardness, fracture resistance, and microleakage [25,26,27].

In a digital workflow, the final device realization through the computer-aided manufacturing (CAM) process can be subtractive or additive. The subtractive method involves milling a monolithic block or disk of a certain material.

In the additive procedures, such as fused deposition, stereolithography, or inject printing, the material is deposited layer by layer to generate the final 3D shape. Currently, 3D-printing has evolved with a wide variety of polymeric materials in order to obtain prosthetic constructions with optimal characteristics [28,29].

This study aims at a comparative analysis of three types of materials that are used for provisional crowns/bridges realization:

- Superpont C + B (SpofaDental, Jicin, Czech Republic), heat-curing acrylic resin,
- Zotion dental milling PMMA block (Zotion, Chongqing, China),
- Freeprint Temp (Detax GmbH & Co. KG, Ettlingen, Germany) resin.

The attention was focused on the analysis of surface roughness before and after finishing and polishing, and the mechanical strength of these materials, following whether there is a significant difference between the devices that were obtained by conventional technology, which uses heat curing resins and the samples that were obtained by subtractive and additive digital technologies.

The null hypothesis in the statistical analysis was that there is no difference between the characteristics of the samples that were obtained by the three types of technologies.

## 2. Materials and Methods

**Figure 1:**
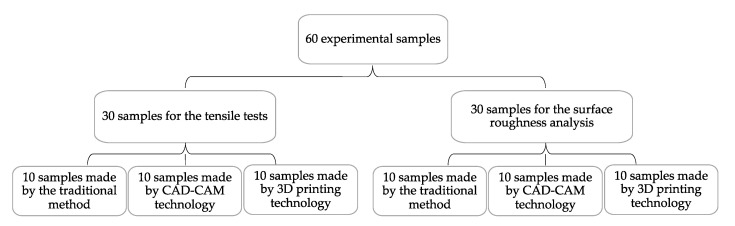
Experimental samples. CAD: Computer-aided design; CAM computer-aided manufacturing.

In order to analyze the characteristics of temporary prostheses materials, we created 60 samples, 30 for the tensile tests and 30 the surface roughness analysis (Figure 1).

A total of 20 samples were made by the traditional method, using the heat-curing acrylic resin Superpont C + B (SpofaDental, Jicin, Czech Republic); 20 were performed by subtractive CAD-CAM technology, using Zotion dental milling PMMA block (Zotion, Chongqing, China); and 20 by additive digital technology, using Freeprint Temp (Detax GmbH & Co. KG, Ettlingen, Germany) resin.

The first step was to make two different shapes of wax patterns. For the traction tests, the wax patterns measured 2 mm thickness with dumbbell-shaped wax patterns with the following dimensions: 75 mm length, 12.5 mm width at the extremities, and 4 mm in the central area. For the roughness tests, the wax patterns were a rectangular shape, also 2 mm thick, 70 mm length, and 30 mm width. The dimensions chosen were in accordance with ASTM D-638 (and ISO 527-2 standards) and were adapted to the requirements of the device that was used in the analysis of the mechanical characteristics.

For the analysis of the surface condition, the sample sizes were also chosen according to the standards that were imposed by the roughness tester that was used.

**Figure 2:**
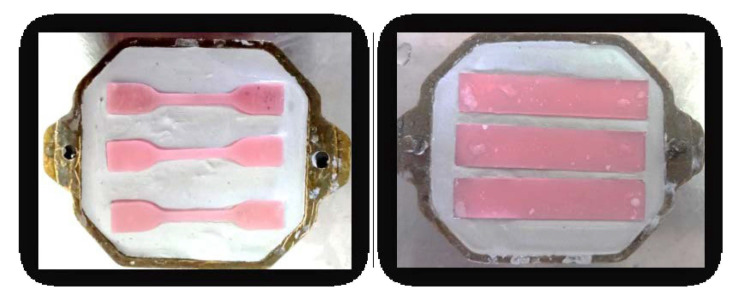
Investing wax pattern and mold realization.

For conventional resin samples the wax patterns, made of pink wax, 2 mm thick (DistriWax-DinstridentPlus, Suceava, Romania) were transformed into acrylic specimens according to the same technology that is used for temporary acrylic dental bridges. They were first invested in dental stone (Elite Rock class IV gypsum-Zhermack, Badia Polesine, Italy) in order to obtain a mold (Figure 2).

After the mold isolation with a separating agent (Isodent/SpofaDental, Jicin, Czech Republic), the acrylic resin was prepared following the producer’s indications: mix 2 g of powder with 1 g of liquid (or in units of volume 3 parts powder to 1 part liquid), the mixing time being 1.5 min.

When the resin has a plastic consistency, it is introduced into the mold and pressed with the help of the hydraulic press. The curing process is performed in a thermo-polymerization chamber at a temperature of 100 °C, pressure 2–4 bar, for 40 min [30,31]. The technological steps are identical to those of the algorithm for making interim bridges from heat-cured resin in dental laboratories.

**Figure 3:**
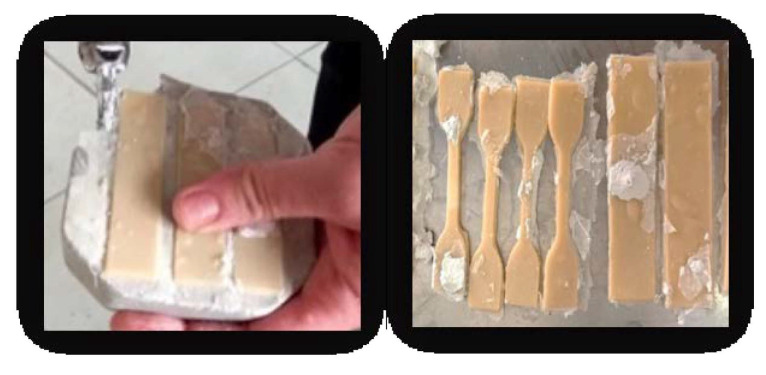
Divesting resin samples.

After cooling, the samples were removed from the mold (Figure 3), verified, and sandblasted.

**Figure 4:**
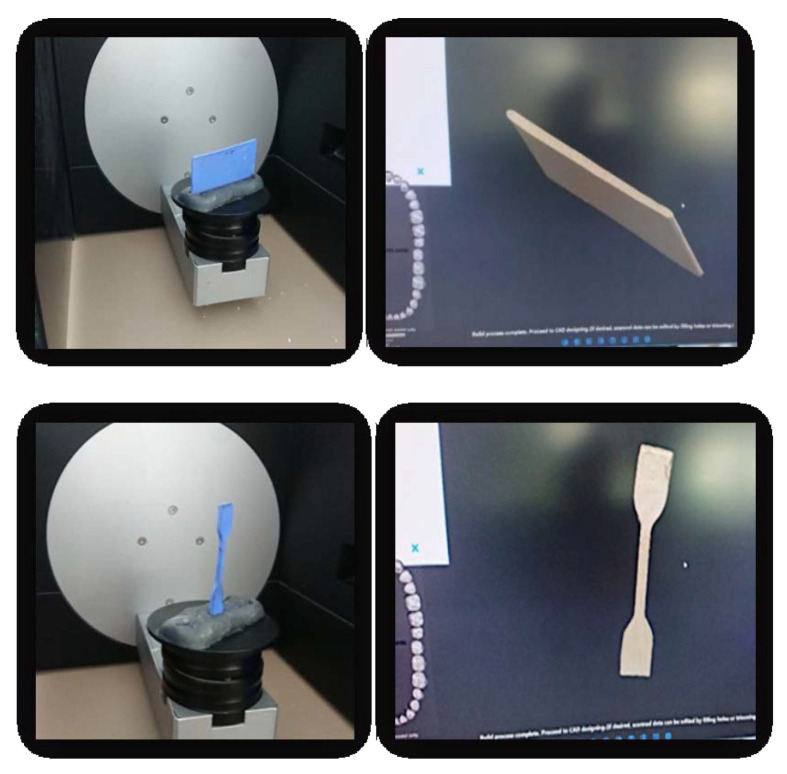
Wax patterns scanning.

Digital samples were realized in a private practice dental laboratory (Draghici Dental, Iasi, Romania). For the subtractive method, the dental milling machine with 5 Axes VHF K5 Plus (VHF, Ammerbuch, Germany) was used. The wax pattern was initially scanned (Swing DOF Scanner, DOF Inc., Seoul, South Korea) in order to obtain their virtual image using the scanning and modeling EXOCAD system (Darmstadt, Germania) (Figure 4).

**Figure 5:**
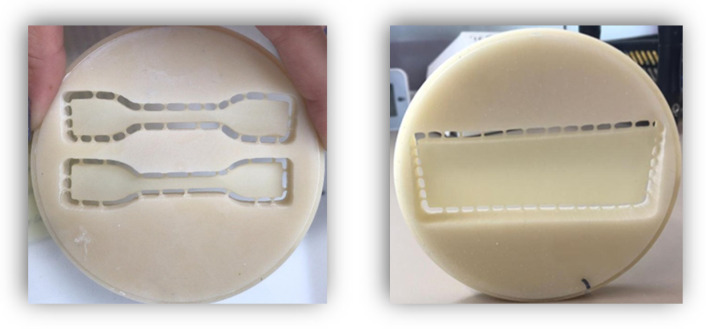
CAD/CAM samples.

A large disk of PMMA A1 monochrome acrylic resin was chosen, with a diameter of 98 mm and a thickness of 20 mm, used for temporary long-term crowns or bridges. The disk was fixed on the plate of the milling machine and based on the information transmitted by the CAD unit, the two types of samples were performed (Figure 5). Then the samples were finished and polished.

**Figure 6:**
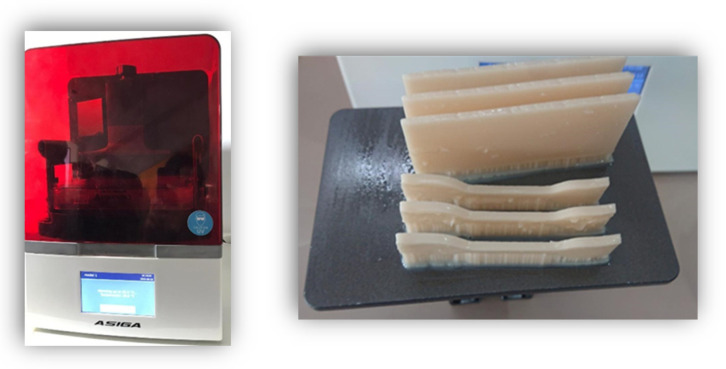
3D Printing samples.

For the additive technology, data on the shape and size of the wax patterns were acquired using the same scanning system and then the information were transmitted to the Asiga MAX 3D printer (Asiga, Alexandria NSW, Australia) (Figure 6).

The printed samples were removed from the build platform, and a hand-piece with a cutting disk was used to separate the supports and the raft from the printed parts. The devices were washed with Isopropyl Alcohol (IPA ≥ 99%)) and dried with the steamer.

The final step was the post-polymerization using Asiga Flash Post Curing Unit (Asiga, Alexandria NSW, Australia), performing a light curing process for 20 min.

All the samples were processed on one surface, according to the same protocol for finishing and polishing temporary bridges, using Acrylic Contouring & Finishing Kit HP (Shofu Dental GmbH, Ratingen, Germany). The surfaces were first adjusted with a dark gray AcryPoint Coarse Grit BP1 tool that was mounted in a hand-piece at low speed (10,000 rpm), followed by finishing with brown AcryPoint Medium Grit BP1 (10,000 rpm). For polishing, a fine AcryPoints (light grey) tool (5000 rpm) was used and soft Circular Goat Hair Brush (90 mm diameter) at a maximum speed of 4000 rpm. For the final gloss we used Buffing Wheel and Universal polishing paste (Ivoclar Vivadent AG, Schaan, Liechtenstein) (4000 rpm).

**Figure 7:**
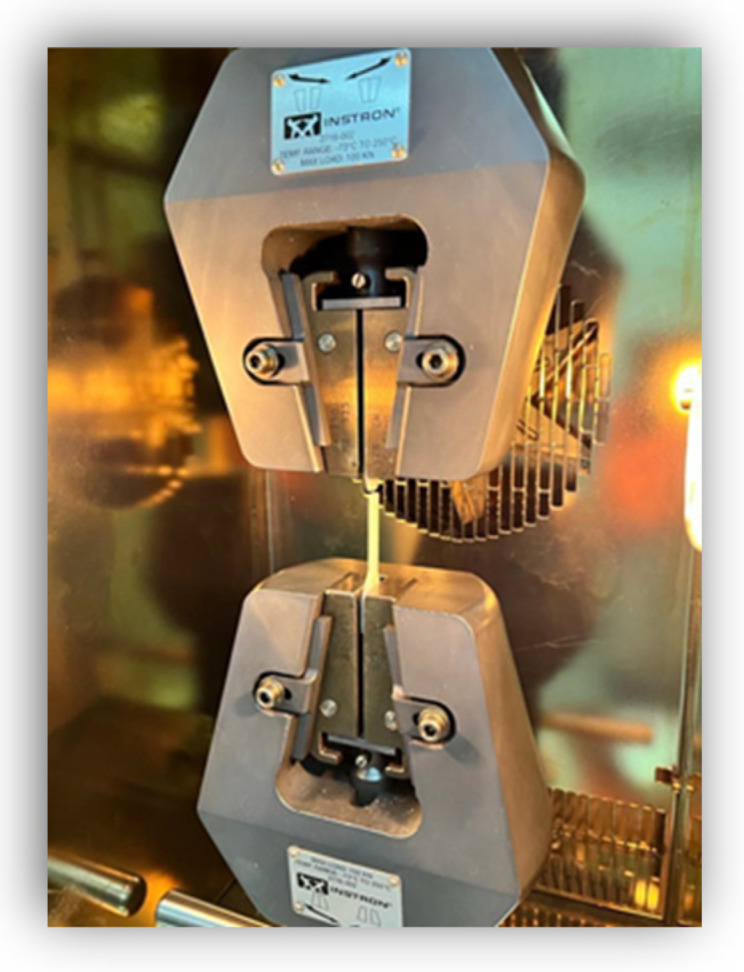
Samples and Instron testing machine.

The mechanical tests were performed in collaboration with Gheorghe Asachi Technical University of Iaşi, Faculty of Materials Science and Engineering. Tensile tests were carried out at room temperature according to the ISO 527-1: 2000 standard, using a computer-controlled testing machine with a dynamic clip-on strain gauge extensometer Dynamic Extensometer Instron 2716-002 (Instron, Norwood, MA, USA) for direct strain measurement. The rectangular specimens were placed and fixed between the grips of the testing machine (Figure 7).

The tensile load was applied at a crosshead speed of 1 mm/min [32]. Young’s modulus (the slope of a secant line between 0.05% and 0.25% strain on a stress-strain plot), tensile yield (tensile stress at yield), and tensile strength (maximum tensile stress during the test) were determined.

To determine the surface roughness, the R_a_, R_z_, and R_q_ parameters were recorded for each sample. R_a_ represents the arithmetical mean of the absolute values of the profile deviations from the mean line of the roughness. R_z_ is the average of all the values that are represented by the maximum height between the maximum and the minimum profile within the assessment length for each sample, and R_q_ represents the root mean square of the surface roughness.

**Figure 8:**
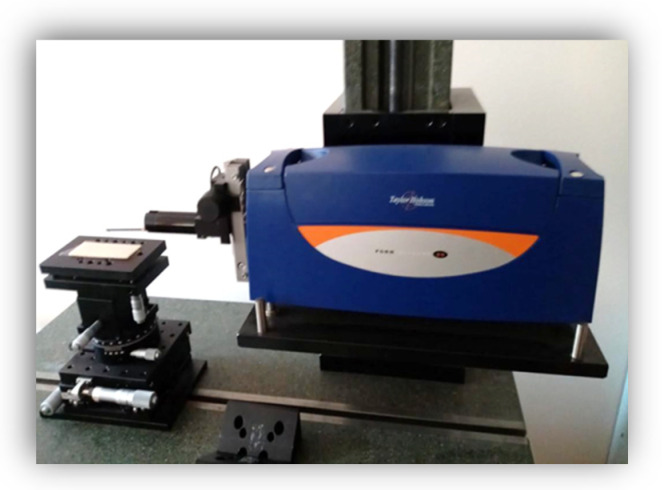
Roughness registration.

A total of three roughness measurements were made on the surface of each sample and the data were recorded with Form Talysurf roughness tester (Taylor Hobson, Leicester, England), whose peak radius of the cantilever is r = 2 µm (Figure 8).

The roughness of the test surfaces was investigated on unfinished and unpolished surfaces and on the finished and polished, surfaces according to the protocol to see if the same processing technique for all the samples, generates significant differences in surface characteristics

The statistical analyses were employed using Stata 16.1 software (StataCorp, College Station, TX, USA). The two-way ANOVA analysis for the two paired samples, before and after the finishing and polishing procedures.

## 3. Results

**Figure 9:**
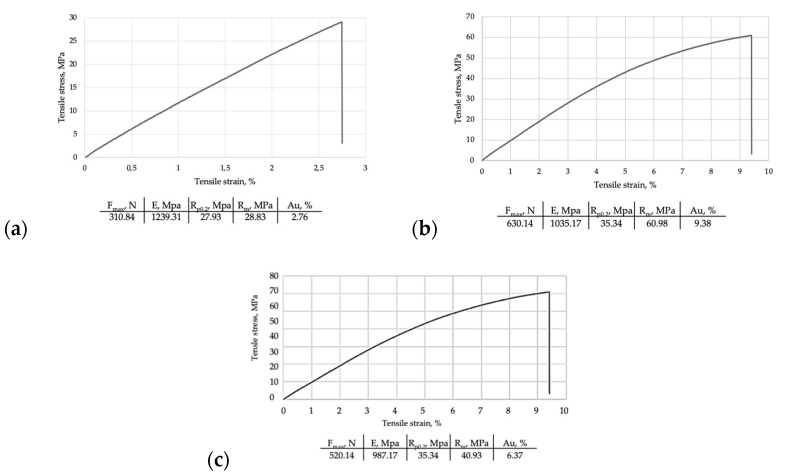
Tensile tests vs. tensile strain diagrams: (**a**) Milled samples; (**b**) 3D printed samples; (**c**) Cured samples.

Tensile stress and tensile strain were calculated for the three categories of samples and the diagrams (Figure 9a–c) illustrate that the best resistance to fracture load has been registered for the milled samples, followed by the heat-cured samples. The lowest value of mechanical resistance was found for the 3D-printed specimens. The tensile behavior of the materials was similar, observing a reversible stage of elastic deformation, followed by an irreversible plastic deformation up to the maximum limit when material fracture occurs.

**Figure 10:**
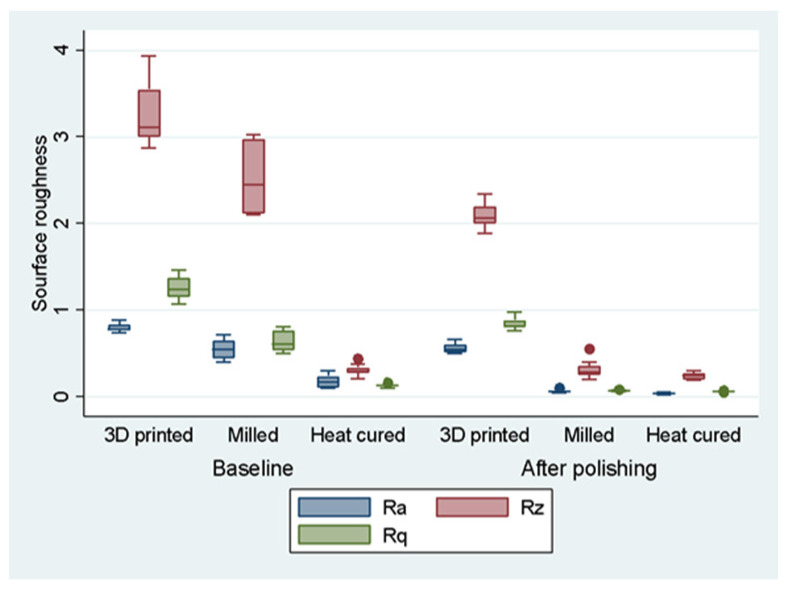
Box plot of surface roughness by resin before/after polishing. Notes: The distribution of rugosity coefficients for each of the three raisins (different colors), both before (baseline) and after polishing are displayed on the Y axis. A total of three measurements for surface rugosity were used: R_a_—arithmetical mean of the absolute values of the profile deviations from the mean line of the roughness; R_z_—average of all values that were represented by the maximum height between the maximum and the minimum profile within the assessment length; R_q_—the root mean square of the surface roughness.

In our study, following the statistical analysis of the results, it can be seen that statistically speaking there are no significant differences between the values of mechanical strength parameters (Figure 10). As such, temporary bridges that are made by digital methods are not significantly different in terms of fracture strength, of those that are performed by conventional methods.

Roughness is an important characteristic of surface quality and can be assessed by determining the micrometric profile of the finished and polished samples.

Surface roughness is quantified by the deviations in the direction of the normal vector of a real surface from its ideal form. If these deviations are large, the surface is rough and if they are small, the surface is smooth. In surface metrology, roughness is typically considered to be the high-frequency, short-wavelength component of a measured surface. However, in practice it is often necessary to know both the amplitude and frequency to ensure that a surface is fit for a purpose.

The shape and dimensions of the micrometric profile have an influence on the adherence and development of the bacterial biofilm at the surfaces of the acrylic prostheses.

The values that were recorded for the surface roughness analysis were centralized to compare the data and establish statistically significant differences. Figure 10 displays an overview of our samples by the three materials that were used (different colors), before and after the polishing procedures. Each sample distribution is reflected by a plot with the following landmarks: the upper and the bottom of the box indicate the values marking the first and third quartiles (Q1 and Q3), the median of the sample is represented by the horizontal line, and the whiskers display the minimum and maximum values of the sample. First, it is interesting to observe that polishing procedures seem to induce a decrease of coefficients for all the three resins. Secondly, the output in Figure 10 suggests that the 3D printed sample displays, on average, the highest coefficients, being followed by the milled and heat-cured ones. The same ranking also applies after polishing. Finally, the highest variation of coefficients is captured when the R_z_ measure is used.

To materialize even more eloquently the differences of the parameters that characterize the surface condition of the three categories of materials, a two way-ANOVA analysis was carried out. In order to check the robustness of our results, the analysis was performed for each of the three rugosity measurements. The normality of data for each sample by treatment, material used, and rugosity measure was initially tested in order to make sure the series were normally distributed. Finally, post hoc multiple comparisons were performed using Tukey’s test.

Table 1 summarizes the results of the two-way ANOVA analysis that was employed on a sample of 30 observations, 10 for each of the materials that was used. The results revealed that surface roughness was significantly influenced by both the type of resin that was used (*p* < 0.001) and the treatment induced by finishing and polishing (*p* < 0.001). Similar *p*-values were obtained for each of the three resins.

It is important to note that there was also a significant interaction between the type of resin that was used and the polishing treatment on the coefficients assessing the surface roughness (R_a_: F(df 2, 54) = 41.46, *p* < 0.001; R_z_: F(df 2, 54) = 97.32, *p* < 0.001 R_q_: F(df 2, 54) = 55.82, *p* < 0.001).

## 4. Discussion

The mechanical characteristics of the materials that are used for prosthetic constructions are influenced by the technological steps. The conventional method that is described in our study, which uses heat-curing resins, involves a large number of laboratory steps, which leads to a longer working time, a longer number of treatment sessions, but also an increased risk of technological errors. On the other hand, this method allows for greater control over the morphology and marginal adaptation of the temporary bridges

Previous clinical studies have yielded conflicting results regarding the effects of fixed interim restorations on periodontal tissues [33,34,35]. However, the conventional wisdom is that fixed interim restorations featuring adequate marginal adaptation and proper finishing and polishing do not induce gingival inflammation, and this was supported by the present results [35].

Digital technologies substantially reduce the workflow, which is a great advantage for both the dental team and the patient. Several studies show that polymers that are used in digital technologies exhibit optimal mechanical parameters compared to conventional PMMA interim resin material. Therefore, these resins are indicated for long-term temporary bridges. The resins for digital technologies are fabricated under controlled industrial conditions and present improved mechanical properties, reduced residual monomers, and, due to the milling fabrication, they show no heat of reaction [36,37,38].

Digholkar et al. [39] compared samples from conventional resins and samples that were obtained by milling and printing methods. The authors concluded that milled samples had the highest flexural strength and 3D-printed samples had the highest microhardness.

The study of Pascutti et. al. [40] emphasized that temporary bridges that were obtained by CAD/CAM methods provided the highest flexural strength values being followed by the heat-curing acrylic resin, which in turn was significantly superior to cold curing resins.

Çakmak.et al. [41] found that the flexural strength of CAD/CAM PMMA-based polymers was higher than the flexural strength of conventional resin.

Pantea et al. [42] observed that the additive manufactured samples exhibited higher elastic moduli (2.4 ± 0.02 GPa and 2.6 ± 0.18 GPa) than the conventional samples (1.3 ± 0.19 GPa and 1.3 ± 0.38 GPa), as well as a higher average bending strength (141 ± 17 MPa and 143 ± 15 MPa) when compared to the conventional samples (88 ± 10 MPa and 76 ± 7 MPa). The results also suggested that the materials were more homogenous when produced via additive manufacturing.

Rayyan et al. [25] showed that that CAD/CAM interim crowns presented stable physical and mechanical properties and may be used for long-term interim restorations.

On the other hand, despite their many benefits, computer technologies are still the most expensive way to make interim crowns and bridges, according to Güth et al. [43].

The results of this study reveal that there are no significant differences between the mechanical characteristics of heat-curing resin samples and those that are obtained by subtractive and additive digital methods.

Other authors reported small differences in fracture force, wear, and roughness between conventional and digital materials for temporary bridges. In vivo and in vitro aging led to comparable results in SEM evaluation. No significant differences in fracture force and wear but differences in roughness, heat of reaction, and thermal weight loss were found in extensive analyses, including simulation of aging processes and mechanical stability [21].

Of course, the strength of temporary bridges is an important quality but not essential for these prosthetic constructions. In contrast, surface characteristics have a much greater impact on the longevity of these prostheses.

Surface roughness enhances plaque retention, promoting bacterial colonization, especially at the restorative margins, resulting in periodontal inflammation and infection. These drastic changes contribute to pulpal sensitivity, gingival recession, tissue inflammation, and complicate the challenge of restorative rehabilitation [44,45,46]. Thus, to all these side effects which would compromise the final therapeutic solution and preserve the periodontal restorative interface, an optimal quality of the interim restoration is desired.

The surface roughness of a restoration alters under the influence of multiple factors, which include the fabrication technique, oral conditions, opposite dentition load, diet, material composition, and polishing techniques [39,47,48]. In the present study, the surface roughness was assessed according to the fabrication technique: CAD/CAM subtractive method, 3D-printing, and the conventional technique. The highest surface roughness was observed in 3D-printing, with a similar outcome to the CAD/CAM milling technique. Nevertheless, the lowest roughness value was observed using the conventional samples. This indicates that conventional heat-cured materials can be successfully used in long-term temporary restoration.

Roughness, especially in the subgingival area, is considered the major cause of plaque buildup and the subsequent inflammatory response. Several sources of roughness have been described: strips and scratches on the surface of carefully polished acrylic resin, separation of the cervical crown margin, and the cervical margin of the finishing line by the luting material exposing the rough surface of the prepared tooth, dissolution and disintegration of the luting material causing crater formation between the preparation and the restoration, and inadequate marginal fit of the restoration [21].

The undersurface of pontics in fixed bridges should barely touch the mucosa and plaque formation determines gingival inflammation and even pseudo pocket formation. The preservation of periodontal health around the crown’s margins is a serious challenge for a dentist and detecting the restoration margin relative to the neighboring bone is a significant factor when providing for the long-lasting health of the gingival tissues [49,50].

The final finishing of the prosthetic restoration affects the development of the microbial biofilm, as increased surface roughness creates a favorable environment for bacterial growth. As such, a prosthetic surface finish from proper manufacturing technique is important [25,27,51]. Most comparative studies have been performed on self-curing resins and resins that are used in digital technologies. Indeed, these resins that are used in the conventional technology, may undergo polymerization contractions, and may have a rougher surface. However, heat-cured resins that were used in our study, allow strict control over each stage, so these drawbacks can be removed. Additionally, further studies are needed in order to also test other materials that are used for provisional prostheses, such as nanofilled composites [52] and fiber-reinforced materials [53].

However, under the conditions of correct preparation of the resin and strict observance of all stages of the polymerization reaction, there is no risk of volumetric changes and the mechanical parameters have optimal values.

Our results confirm that there is a statistically significant difference in terms of surface condition between the baseline values and the values after polishing (*p* < 0.001). The statistically significant difference was confirmed for each of the three resins, with similar *p*-values.

This is in line with the results that were suggested by the descriptive statistics, which revealed a sizeable downward trend. The same result holds true for all the three resins that were investigated in our study. For the heat cured sample, the difference is only statistically significant when looking at the R_a_ coefficients, but not for the other two measurements. The sharpest reduction is displayed by the milled resin which displayed average reductions of more than 44% for each of the three measurements that were investigated. It is also interesting to add that the highest coefficients values are shown, on average, in the 3D-printed samples. The means for this material was also confirmed to be statistically different from the means that were displayed by the other two resins that were used. The same result hold true after the polishing process. At the other end lies the heat-cured samples which reports the lowest averages.

It was shown that CAD/CAM temporary restorations have superior mechanical properties and superior surface quality compared with their conventional counterparts [54,55,56,57], as evidenced by our study.

The temporary prosthesis should not be considered as a useless stage with uncertain indications but as a way of transition from disability to functionality. The materials and methods which are used to make interim bridges are of a major importance for achieving this type of prosthesis.

The direct technique in the dental office is an alternative which is used in practice for temporary bridge fabrication. In this technique, the patient undergoes interim prosthesis in the same stage as the abutment preparation, which is an advantage because intermediate laboratory procedures are eliminated. However, the direct technique has significant disadvantages such as poorer marginal fit, pulpal damage due to the temperature released by the resin polymerization reaction, lower mechanical strength, and inaccurate dental morphological rehabilitation. Therefore, the routine use of directly formed interim restoration is not recommended when indirect techniques (conventional or digital) are feasible [36,58].

One of the study’s limitations includes a limited number of materials and mechanical tests investigated. However, we believe it is essential to examine these three types of resins, which are commonly used in dental laboratories. Further research is needed over a wider range of materials and possibly extending it with other methods of analysis that more accurately reflect the mechanical parameters.

The fact that the printed samples were obtained exclusively through digital light processing is another limitation. Future studies should focus on the behavior of samples that are made with stereolithography (SLA) and material jetting (Polyjet).

## 5. Conclusions

Within the limitations of this study, we can conclude that temporary bridges that are made using the subtractive technology can allow obtaining resistant devices with low roughness structure. At the same time, we can ensure an optimal restoration of the teeth morphology and a correct functional rehabilitation.

Therefore, these methods can be used to make long-term interim prosthodontics restorations.

In this stage of the research, we focused on the analysis of some frequently used resins in the dental laboratory for temporary bridges realization. In order to obtain even more relevant results, the study will be continued, taking into account other resins that are used for these prosthetic constructions.

In the current era, conventional methods using heat-curing resins are still a viable alternative and these materials can be successfully used for short-term temporary prosthetic restorations.

## Figures and Tables

**Table 1:** Means (±SEM) of surface roughness parameters by resin that was used before and after polishing.

Surface Roughness	3D Printed			Milled			Heat Cured		
	Baseline	After Polishing	ΔR%	Baseline	After Polishing	ΔR%	Baseline	After Polishing	ΔR%
R_a_	0.80(0.02) ^a^	0.56(0.02) ^b^	−15.10	0.55(0.04) ^b^	0.06(0.00) ^d^	−44.61	0.17(0.02) ^c^	0.04(0.00) ^d^	−38.78
R_z_	3.26(0.12) ^a^	2.08(0.04) ^c^	−18.07	2.51(0.13) ^b^	0.32(0.03) ^d^	−43.73	0.31(0.02) ^d^	0.24(0.01) ^d^	−10.28
R_q_	1.26(0.04) ^a^	0.86(0.02) ^b^	−15.75	0.64(0.04) ^c^	0.07(0.00) ^d^	−44.60	0.13(0.00) ^d^	0.06(0.00) ^d^	−26.51

Note: n = 10 obs. per sample ^a–d^ Means in a row without a common superscript letter are different (*p* < 0.05) as evidenced by two-way ANOVA and the Tukey’s test. R_a_: arithmetical mean of the absolute values of the profile deviations from the mean line of the roughness; R_z_: average of all values that are represented by the maximum height between the maximum and the minimum profile within the assessment length; R_q_: the root mean square of the surface roughness.

## Data Availability

Not applicable.
